# Characterization of 3 Megabase-Sized Circular Replicons from *Vibrio cholerae*

**DOI:** 10.3201/eid2107.141055

**Published:** 2015-07

**Authors:** Kazuhisa Okada, Wirongrong Natakuathung, Mathukorn Na-Ubol, Amonrattana Roobthaisong, Warawan Wongboot, Fumito Maruyama, Ichiro Nakagawa, Siriporn Chantaroj, Shigeyuki Hamada

**Affiliations:** Thailand–Japan Research Collaboration Center on Emerging and Re-emerging Infections, Nonthaburi, Thailand (K. Okada, W. Natakuathung, M. Na-Ubol, A. Roobthaisong, W. Wongboot, S. Hamada);; Osaka University, Osaka, Japan (K. Okada, S. Hamada);; Tokyo Medical and Dental University, Tokyo, Japan (F. Maruyama, I. Nakagawa);; National Institute of Health, Nonthaburi (S. Chantaroj)

**Keywords:** Vibrio cholerae, cholera, bacteria, Thailand, virulence, replicons

**To the Editor:** Prokaryotes typically have a single circular chromosome. However, some bacteria have >1 chromosome. *Vibrio* bacteria, for example, have 2 circular chromosomes: 1 (Ch1) and 2 (Ch2) (*1*–*3*). Most recognizable genes responsible for essential cell functions and pathogenicity are located on Ch1. Ch2 is also thought to encode some genes essential for normal cell function and those associated with virulence. Both chromosomes are controlled coordinately in their replication and segregation (*4*). Evidence suggests that Ch2 was originally a mega-plasmid captured by an ancestral *Vibrio* species (*2**,**5*). We report the characterization of recent isolates of *V. cholerae* O1 from Thailand that carry a novel gigantic replicon (Rep.3) in addition to Ch1 and Ch2.

Cholera outbreaks occurred in Tak Province, Thailand, during March–December 2010. We obtained 118 isolates of *V. cholerae* O1 and subjected their *Not*I digests to pulsed-field gel electrophoresis (PFGE), which differentiated the isolates into 8 different patterns (*6*). The profile of PFGE type A6 was identical to that of PFGE type A4, except that a large DNA band existed in type A6. The PFGE profile of the intact (undigested) DNA of the type A6 isolates exhibited a unique genome structure consisting of 3 large replicons (Figure).

**Figure:**
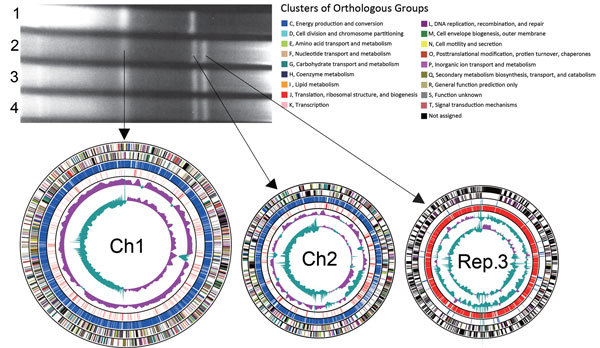
Pulsed-field gel electrophoresis of intact genomic DNA of *Vibrio cholerae* isolates and circular representation of the genome of *V. cholerae* O1 El Tor TSY216, consisting of 3 chromosomes. The preparation of genomic DNA embedded in agarose gels and the protocol for pulsed-field gel electrophoresis have been described previously (*5*). Arrows indicate DNA bands that correspond to the chromosomes. Lanes: 1, N16961 reference strain carrying Ch1 (2.96 Mb) and Ch2 (1.07 Mb) (*2*); 2, TSY216; 3, TSY241; 4, TSY421. The first and second outermost 2 circles of each schematic chromosome show the COGs, functional categories of the coding regions of TSY216, in the clockwise and anticlockwise directions, respectively. The third and fourth outermost circles of each schematic chromosome show the coding sequence-assigned (blue) and coding sequences-unassigned (red) functions of the products, respectively. The third and fourth circles show the GC content of the TSY216 sequence and the percent G+C deviation according to the strand, respectively. Ch1, chromosome 1; Ch2, chromosome 2; COGs, clusters of orthologous groups of proteins; Rep.3, novel replicon.

Three isolates of PFGE type A6 (TSY216, TSY241, and TSY421) were obtained during June 3–July 5, 2010, from 3 unrelated residents of a village near the Thailand–Myanmar border. The isolates were classified as multilocus variable-number tandem-repeat analysis type 16, suggesting that they are of clonal origin (*6*). Next, we performed whole-genome sequencing of TSY216, as a representative of PFGE type A6 isolates, by using the GS FLX Titanium system (8 kb–span paired-end library; Roche, Indianapolis, IN, USA). Using Newbler version 2.6, the Roche 454 GS De Novo Assembler software (454 Life Sciences, Branford, CT, USA), we assembled 424,273 reads into 3 large scaffolds comprising 119 contigs at 18.3-fold coverage. The gaps between contigs were closed by PCR, and the PCR products were then sequenced. Illumina sequence data (Illumina, Inc., San Diego, CA, USA) were used to improve low-quality regions. The whole-genome sequence of TSY216 was completed and deposited in GenBank (accession nos. CP007653–55).

Full-genome sequencing revealed that *V. cholerae* O1 El Tor TSY216 consists of 3 circular replicons, Ch1 (3,053,204 bp), Ch2 (1,051,284 bp), and Rep.3 (896,006 bp), with an average G+C content of 47.7%, 47.0%, and 37.3%, respectively. In total, 4,579 coding sequences were detected and annotated by using the National Center for Biotechnology Information Prokaryotic Genome Annotation Pipeline (http://www.ncbi.nlm.nih.gov/genome/annotation_prok/). The whole-genome comparison between 2010EL-1786 (an outbreak isolate from Haiti) (*7*) and TSY216 revealed that Ch1 and Ch2 shared nearly identical gene content and showed conserved synteny, but integrative and conjugative elements were distinguishable. Strain TSY216 carries CTX-3, whereas strain 2010EL-1786 possesses CTX-3b. These CTXs represent wave 3 of the seventh cholera pandemic (*8*). Rep.3 of TSY216 did not share a conserved region with Ch1 and Ch2. Thus, this replicon may have been gained fairly recently through horizontal gene transfer from unknown organisms.

Rep.3 encodes 999 coding sequences and 66 transfer RNAs, among which 39 have been assigned putative functions and 960 encode hypothetical proteins and proteins of unknown function. The origin of the replicon could not be traced from the coding sequences in the public databases. Of note, Rep.3 encodes a specific transfer RNA for each amino acid, for a total of 20 amino acids. In addition, Rep.3 carries 2 genes encoding the histone-like nucleoid-structuring protein. In this regard, a 165-kb plasmid, pSf-R27, in *Shigella flexneri* encodes a histone-like nucleoid-structuring protein that was claimed to be a transcriptional repressor of the plasmid (*9*). Rep.3 may have a stealth strategy similar to that of pSf-R27.

We assessed the stability of the Rep.3 of the 3 A6 isolates. In total, 96 colonies for the 3 isolates were subcultured each day for 30 consecutive days. Then, using PCR and PFGE, we determined whether Rep.3 remained in the 96 subcultures. The Rep.3-specific primer set (Rep3hns-F: 5′-TTCAATGCGTCCAGCGTTGC-3′ and Rep3hns-R: 5′-TCGCACCTCTATCAATAGCC-3′) for PCR was designed for detection of the histone-like nucleoid-structuring protein gene encoded on the third replicon. All subcultures maintained Rep.3 in an unchanged state. However, when the organisms were cultured at 42°C, ≈70% of the subcultures lost Rep.3. The growth rates of the organisms with and without Rep.3 showed no substantial difference when the organisms were cultured in Luria-Bertani medium at 37°C.

The appearance of *V. cholerae* O1 variants with additional circular replicons may contribute to evolution of the bacteria in unexpected manners. Clones from the seventh cholera pandemic, which began in 1961, share nearly identical gene content (*8**,**10*). However, some clones, such as TSY216, can gain a replicon of megabase class and maintain it stably. Eventually, epidemic *V. cholerae* O1 may gain the ability to incorporate genes that change properties such as antigenicity or pathogenicity. The function of Rep.3 remains under investigation.
